# New medicines to improve control and contribute to the eradication of malaria

**DOI:** 10.1186/1475-2875-9-S2-I15

**Published:** 2010-10-20

**Authors:** Timothy NC Wells

**Affiliations:** 1 Medicines for Malaria Venture, 20 Route de Pré-Bois, 1215, Geneva, Switzerland

## 

Despite being one of the most prevalent tropical diseases, for many years malaria was not a commercial priority for the pharmaceutical industry. However, in response to the emergence and spread of resistance to the available antimalarial drugs, there has been a renaissance in the discovery and development of new medicines to control the disease in the last few years. The persistent threat of resistance means that new molecules with novel mechanisms of action are continually required. Furthermore, the recent call for the elimination and eradication of malaria has prompted an extension of the stages of the life cycle of malaria parasites that should be targeted by new molecules. Recent advances in genome-based technologies and *in vitro* screening of whole parasites have broadened the range of therapeutic targets and are accelerating the development of a new generation of treatments for both malaria control and eradication.

